# Frequent reduction or loss of DCC gene expression in human osteosarcoma.

**DOI:** 10.1038/bjc.1997.222

**Published:** 1997

**Authors:** M. A. Horstmann, M. PÃ¶sl, R. B. Scholz, B. Anderegg, P. Simon, K. Baumgaertl, G. Delling, H. Kabisch

**Affiliations:** Department of Paediatric Haematology/Oncology, University Hospital Eppendorf, Hamburg, Germany.

## Abstract

**Images:**


British Journal of Cancer (1997) 75(9), 1309–1317
© 1997 Cancer Research Campaign

### Frequent reduction or loss of *DCC* gene expression in human osteosarcoma

**MA Horstmann[1], MPösl[2], RB Scholz[1], B Anderegg[1], P Simon[1], K Baumgaertl[2], G Delling[2] and H Kabisch[1]**

Departments of[1] Paediatric Haematology/Oncology, and Osteopathology, University Hospital Eppendorf, 20246 Hamburg, Germany

**Summary** The 'deleted in colon carcinoma' (*DCC*) gene has been considered a candidate tumour-suppressor gene that encodes for a transmembrane protein with strong structural similarity to members of the superfamily of neural cell adhesion molecules. It has been mapped to the chromosomal region 18q21.1 and it is implicated in cellular differentiation and developmental processes. In human osteosarcoma allelic loss frequently occurs on the long arm of chromosome 18, suggesting a possible involvement of the *DCC* gene in the pathogenesis of this tumour entity. In the present study the mRNA and protein expression and rearrangements at the DNA level of the *DCC* gene were addressed in 25 osteosarcomas and several tumour cell lines, including osteosarcoma- and colon carcinoma-derived cell lines. Using an reverse transcriphase polymerase chain reach in (RT-PCR)-based approach *DCC* expression was found to be lost or substantially reduced in 14 of 19 high-grade osteosarcomas, in three of six lower grade osteosarcomas and most of the tumour cell lines, in contrast to normally differentiated osteoblasts. Immunohistochemical studies on DCC protein expression of 14 selected tumours correlated well with the RT-PCR-based results. In view of the putative tumour-suppressor characteristics of the *DCC* gene its loss or reduction of expression could be a specific event in the development or progression of many high-grade osteosarcomas.

**Keywords:** osteosarcoma; *DCC* gene; tumour suppressor

Consistent chromosomal losses in human malignancies often imply that the affected region may contain a tumour-suppressor gene. In osteosarcomas frequent allelic deletions have been detected at chromosome arms 3q, 13q, 17p and 18q, the latter of which has been found to be affected in 7 of 11 investigated tumours (Yamaguchi et al, 1992). In 1990, a putative tumour-suppressor gene was identified and has been mapped to the chromosomal region 18q21.1. Based on studies on allelic delections in colorectal cancer it has been termed 'deleted in colon carcinoma (*DCC*) gene' (Fearon et al, 1990).

Its open reading frame comprises 29 exons that encode for a transmembrane neural cell adhesion molecule consisting of four immunoglobulin-like and six fibronectin type III-like extracellular domains and a still poorly characterized intracytoplasmic domain (Cho et al, 1994). In colorectal cancer a homozygous deletion, point mutations and DNA insertions have been observed within the *DCC* gene. Moreover, loss of heterozygosity (LOH) and reduction or loss of expression of the *DCC* transcript are common in prostate, breast, oesophageal, endometrial, glial and germ-cell cancer, as well as some haematological malignancies (Cropp et al, 1990; Hohne et al, 1992; Uchino et al, 1992; Gao et al, 1993; Miyake et al, 1993; Porfiri et al, 1993; Scheckr Coons, 1993; Thompson et al, 1993).

Results of functional in vitro studies such as cytomegalovirus expression vector-mediated *DCC* cDNA transfection experiments or antisense RNA strategies to *DCC* endorse its role as a tumour-suppressor gene (Narayanan et al, 1992; Klingelhutz et al, 1995). The *DCC* gene has been implicated in terminal cellular differentiation and developmental processes, perhaps through control of cell–cell or cell–extracellular matrix interactions. Like other cell adhesion molecules *DCC* is expressed at the cell surface but at relatively low levels, thus suggesting that its role may rather be that of a signal-transducing receptor than an anchorage protein mechanically preserving cellular texture (Hedrick et al, 1994). Its loss may confer a growth advantage on evolving cells.

In human osteosarcoma the frequently observed loss of heterozygosity at chromosome arms 13q and 17p may involve the retinoblastoma and p53 tumour-suppressor genes (Friend et al, 1986; Toguchida et al, 1989; Miller et al, 1990; Mulligan et al, 1990) whereas the candidate genes within the LOH loci at chromosome arms 3q and 18q are still undefined. The localization of the *DCC* gene at the chromosomal region 18q21.1 may suggest its involvement in the pathogenesis of this tumour entity. To prove this hypothesis we assessed 25 specimens of human osteosarcoma from 22 patients and various osteosarcoma- and other childhood sarcoma-derived cell lines with regard to mRNA and protein expression and DNA rearrangement of the *DCC* gene. Various normally differentiated mesenchymal tissues, including osseous specimens and several colon carcinoma cell lines with known expression patterns of the *DCC* gene (Fearon et al, 1990) were included in the study.

### MATERIALS AND METHODS

### Tissue specimens

Twenty-five human osteosarcomas were obtained from 22 patients who underwent surgery at the University Clinic Hamburg-Eppendorf or associated treatment centres of the Cooperative Osteosarcoma Study Group (Winkler et al, 1988). The selection of

*Received 22 July 1996*
*Revised 3 October 1996*
*Accepted 16 October 1996*

*Correspondence to:* MA Horstmann

Presented in part at the AACR meeting 'Cancer – the interface between basic and applied research', Baltimore, 5–8 November 1995.

**Table 1** Clinicopathological data

| OS no. | Patient No. | Age at diagnosis (years) | Primary tumour | Specimen | Histological subtype (grading) | Chemotherapy[a] before sampling | Admixture of non-malignant cells |
|---|---|---|---|---|---|---|---|
| 1 | 1 | 22 | Femur | p | High-grade chondroblastic | + | (–) |
| 2 | 2 | 14 | Femur | p | High-grade osteoblastic | + | (–) p[b] |
| 3 | 3 | 17 | Femur | m (lung) | High-grade chondroblastic | – | (–) |
| 4 | 4 | 52 | Femur | p | High-grade osteoblastic | – | (–) |
| 5[c] | 5 | 15 | Femur | m (lung) | High-grade osteoblastic | – | (–) |
| 6 | 6 | 33 | NE | m (spine) | High-grade osteoblastic | + | (–) |
| 7 | 7 | 16 | Tibia | p | High-grade osteoblastic | + | (–) |
| 8 | 8 | 18 | Femur | m (lung) | High-grade osteoblastic | – | (–) |
| 9 | 9 | 14 | Humerus | p | High-grade osteoblastic | + | (–) |
| 10 | 10 | 8 | Humerus | p | High-grade osteoblastic | + | 10–15% |
| 11 | 11 | 9 | Secondary OS after RB | p | High-grade osteoblastic | + | (–) |
| 12 | 12 | NE | Femur | m (lung) | High-grade osteoblastic | – | (–) |
| 13 | 13 | 20 | Femur | p | High-grade chondroblastic | + | (–)[b] |
| 14 | 14 | 15 | Femur | p | High-grade chondroblastic | + | <10% |
| 15 | 15 | 85 | NE | p | High-grade osteoblastic | – | (–) |
| 16 | 16 | 12 | Fibula | p | High-grade osteoblastic | + | (–) |
| 17 | 17 | 13 | Tibia | p | High-grade osteoblastic | + | (–) |
| 18 | 18 | 13 | Fibula | p | High-grade chondroblastic | + | (–) |
| 19[c] | 5 | 15 | Femur | m (lung) | High-grade osteoblastic | – | (–) |
| 20 | 19 | 11 | Tibia | p | Intermediate periosteal | – | (–) |
| 21 | 19 | 11 | Tibia | r | Intermediate periosteal | – | (–) |
| 22 | 20 | 7 | Femur, secondary OS after RMS | p | Intermediate periosteal | – | (–) |
| 23 | 21 | 16 | Os ileum | p | Low-grade | – | (–) |
| 24 | 22 | 17 | Humerus | r | Low-grade | – | (–) |
| 25 | 22 | 17 | Humerus | m (lung) | Low-grade | – | (–) |

[a]According to the guidelines of the Cooperative Osteosarcoma Study (COSS 86) (Winkler et al, 1988). p, primary tumour m, metastasis; r, relapse; RB, retinoblastoma; RMS, rhabdomyosarcoma; NE, not evaluable; (–) absence of significant amounts of non-malignant cells except for stroma cells. [b]Approximately 20% vital tumour cells and 80% necrosis. [c]Metachronous lung metastases occurring 7 and 8 years after primary disease respectively.

**Table 2** Cell lines

| | |
|---|---|
| Osteogenic sarcoma | TE85, Saos-2, U-2 OS, KHOS-240S, Wo-OS |
| Colorectal adenocarcinoma | SW48, SW403, SW948, SW1116, SW1463, HCT116 |
| Rhabdomyosarcoma | A-204 |
| Ewing's sarcoma | RD-ES |
| Acute T-lymphoblastic leukaemia | CCRF-CEM |

cases was based on the availability of frozen tissue material, which was stored at –80°C, as well as paraffin-embedded tissue specimens. Each tumour specimen was thoroughly evaluated by microscopic examination, particularly with regard to vitality of tumour cells and contamination with non-malignant cells. Twenty-one out of 25 tumours consisted almost exclusively of vital tumour cells except for an insignificant admixture of stroma cells. As shown in Table 1 12 tumours were obtained after preoperative chemotherapy. Nine of these were non-responsive and contained no significant normal cell populations. Two tumours (OS10, 14) contained minor amounts of granulation tissue, whereas two others (OS 2,13) revealed considerable necrosis. Tumours revealing more than 90% chemotherapy-induced necrosis or more than 20% non-malignant vital tissue were excluded from the study. Clinical and pathological data are summarized in Table 1. In cases OS11 and OS22, a retinoblastoma and a rhabdomyosarcoma respectively had preceded the development of osteosarcoma. In three cases two different tumour specimens were collected from one patient each as indicated by patient numbers in Table 1. Specimens OS5 and OS19 (patient no.5) were metachronous lung metastases of a high-grade osteosarcoma that occurred 7 and 8 years after primary disease respectively. Patient no. 19 initially presented with an intermediate-grade periosteal osteosarcoma (OS20), which relapsed 4 months after limb-salvaging surgery (OS21). OS24 and OS25 represent a second local relapse of a low-grade osteosarcoma and a concomitant lung metastasis, both of which occurred nearly 2 years after the initial diagnosis.

As a control normal bone tissue devoid of adherent bone marrow cells was obtained from 11 patients undergoing joint replacement procedures because of non-inflammatory and non-malignant joint disease. One case of myositis ossificans was included to demonstrate differential *DCC* expression in undifferentiated mesenchymal cells and mature osteoblasts/ osteocytes. Additionally, paraffin-embedded specimens of granulation tissue from chemotherapy-responsive osteosarcomas were included as controls. For RNA studies matching of osteosarcoma and normal bone tissue in each patient was not feasible because normal bone tissue was not collected prospectively.

### Cell lines

Table 2 gives an overview of the origin of cell lines used in this study. All cell lines were of human origin and were purchased from the American Type Culture Collection (Rockville, MD, USA) except for one osteogenic sarcoma derived-cell line referred to as Wo-OS, which was established in our institution. Osteosarcoma cell lines Saos-2, U2-OS, Wo-Os, KHOS-24OS and the rhabdomyosarcoma

 © Cancer Research Campaign 1997

cell line A-204 were grown in McCoy's 5a medium supplemented with 10% fetal calf serum (FCS). The osteosarcoma cell line TE85 and the Ewing's sarcoma cell line RD-ES, as well as the T-ALL cell line CCRF-CEM, were grown in RPMI-1640 with 10–15% FCS. Colon carcinoma cell lines HCT 116, SW 1463, SW 403, SW 948, SW 48 and SW 1116 were cultured in MEM/RPMI-1640 (ratio 3:1) supplemented with 10% FCS.

### Total RNA isolation and DNA extraction

Osteosarcoma tissue was submerged in liquid nitrogen and subsequently ground to powder by a microdismembrator (Braun Biotech Int.). Normal cortical bone specimens were smashed to small fragments and subsequently washed twice in Hanks' solution for 30 s to remove adherent non-osseous cells. Cells were lysed in guanidine–thiocyanate buffer. DNA and total RNA were isolated after ultracentrifugation on a caesium chloride cushion (Chirgwin et al, 1979). In cases of insufficient yield of total RNA we used a monophasic solution of phenol and guanidine–thiocyanate (Chomczynski and Sacchi, 1987).

### DNA blot analysis

An aliquot of 10 μg of *Eco*RI-digested genomic DNA was electrophoresed on a 0.8% agarose gel and transferred to a Gene Screen Plus nylon membrane (DuPont). Nylon filters were hybridized with a [$^{32}$P]-CTP-labelled *DCC* 1.65kb cDNA probe comprising nucleotides 591–2250 (exons 3–15) of the *DCC* cDNA sequence for 18 h at 65°C. Washes and autoradiography were performed as described previously (Maniatis et al, 1989).

### Reverse transcriptase- polymerase chain reaction (RT-PCR)

An aliquot of 1 μg of total RNA was reverse transcribed. The synthesis of the first strand cDNA was primed with random hexamers performed at 42°C for 1 h using 200 units M-MLV reverse transcriptase (Promega, Madison, WI, USA). After RNAase H treatment of the RNA/DNA hybrid, one half of the cDNA was used for PCR amplification in a 100-μl reaction mix containing 50 pmol of each primer, 2.5 U of *Taq* polymerase (Boehringer, Mannheim), 200 μmol $l^{-1}$ dNTPs, 10 mmol $l^{-1}$ Tris-HCL pH 8.0, 50 mmol $l^{-1}$ potassium chloride, 1.5 mmol $l^{-1}$ Magnesium chloride. *DCC* complementary DNA was amplified under the following conditions: 94°C for 30 s; 58°C for 75 s; 72°C for 30 s for 35 cycles. The initial denaturation was performed at 95°C for 1 min. For the final extension, temperature was held at 72°C for 5 min. *DCC*-specific primers were designed that span exons 5–6 of the *DCC* cDNA sequence (nt 986–1218) coding for the extracellular immunoglobulin-like domains no. 3 and 4 of the DCC protein. Owing to this exon connection strategy, DNAase I treatment of the initial template had no effect on the resulting PCR products. To confirm the integrity of the RNA used to generate the cDNAs and to check for equivalent efficiency of amplification all RT-PCR experiments were performed using sets of primers specific for the β-actin as well as the glucose 6-phosphate dehydrogenase (G6PD) housekeeping genes (Persico et al, 1981; Nakajima-Iijima et al, 1985; Adams et al, 1992), which are located on chromosomes X and 7 respectively. Both genes code for highly conserved proteins. β-Actin is found in abundance in eukaryotic cells, whereas G6PD is normally expressed at a very low level accounting for less than 0.1% of total RNA. RT-PCR experiments were done as a biplex PCR, i.e. PCR reactions were run containing the *DCC*-specific primers combined with *β-actin* or *G6PD* specific sets of primers. To exclude an interference of target and control primers resulting in a reduced rate of amplification of either gene product the volume of the RT reaction was split and the cDNAs were amplified separately with the *DCC* and control primers respectively. PCR products were sampled during the exponential phase of amplification, as could be demonstrated for *DCC*-specific primers as well as controls (data not shown). The RT-PCR experiments were finally carried out twice as a biplex PCR for each sample to check for reproducible results. Concentrations of control primers were 25 pmol $l^{-1}$ each; experiments were performed under the above-mentioned conditions. Primers used were

5′-TTCCGCCATGGTTTTTAAATCA-3′ (*DCC* sense),
5′-AGCCTCATTTTCAGCCACACA-3′ (*DCC* antisense) (Fearon et al, 1990),
5′-ATTCATCATCATGGGTGCATCG-3′ (G6PD sense),
5′-TGTTTGCGGATGTCAGCCACTGT-3′ (G6PD antisense),
5′-TGCTATCCAGGCTGTGCTAT-3′ (actin sense),
5′-GATGGAGTTGAAGGTAGTTT-3′ (actin antisense).

In order to examine the specificity and the relative amount of the generated PCR product one-fifth (20 μl) of each PCR reaction was electrophoresed and blotted onto a nylon membrane by capillary salt transfer using a 10×SSC solution (1×SSC=0.15 M sodium chloride/0.015 sodium citrate). Blots were hybridized with $1\times10^6$ c.p.m. of the [$^{32}$P]CTP-labelled 1.65 *DCC* cDNA probe per ml at 65°C for 16 h. After hybridization blots were washed twice with 0.1×SSC/0.1% sodium dodecyl sulphate (SDS) at 60°C for 30 min and autoradiographed at 4°C for 12 h. *DCC* RT-PCR products from normal bone tissue were included in each set of probes as a positive control. Furthermore, after stripping off the *DCC* cDNA probe the co-amplified β-actin-specific PCR products were hybridized with $0.5 \times 10^6$ c.p.m. per ml of a $^{32}$P-labelled actin probe.

An aliquot of μg of total RNA from each normal bone specimen was reverse transcribed and the prepared cDNAs were serially diluted from 1:1 to 1:1000, and subsequently amplified under conditions identical to those described above.

### RT–PCR data analysis

The intensity of individual autoradiographic signals of blotted PCR products was measured by densitometry scanning using a Shimadzu densitometer. The area under the curve of absorption (AUC of A) was calculated with computer assistance. In order to estimate the relative abundance of *DCC* transcripts, values were compared with the mean AUCs resulting from the serially diluted cDNAs from normal bone specimens (Figure 1).

### Preparation of cell line and tissue protein lysates

Cell homogenates were solubilized in Tris-buffered saline [25 mM Tris (hydroxyureaethylaminomethane), pH 8] with detergents (1% deoxycholate, 1% Nonidet p-40, and 0.1% SDS) and protease inhibitors (50 μg $ml^{-1}$ antipain, 5 μg $ml^{-1}$ leupeptin, 100 μg $ml^{-1}$ phenylmethylsulfonyl fluoride, and 1 mM EDTA). Protein concentrations were determined photometrically after adjustment with known albumin concentrations.

© Cancer Research Campaign 1997

**Figure 1** PCR amplification yields of serially diluted cDNAs from normal bone tissue. An aliquot of 1 µg of RNA from 11 normal osseous tissue specimens was reverse transcribed. Each cDNA was diluted from 1:1 to 1:1000 and thereafter amplified under identical conditions. The graph illustrates the relationship between the mean absorption of autoradiographic signals (including standard deviation) and the magnitude of cDNA dilution shown as its logarithm. Note the maximum of amplification at a *DCC* cDNA dilution of about 1:2

### Immunoprecipitation and immunoblot analysis of *DCC* expression

Unprocessed cell line and tissue lysates were precleared by incubation with purified mouse immunoglobulin and protein G/Sepharose (Pharmacia, Germany). The supernatant was incubated with protein G/Sepharose and *DCC*-specific monoclonal antibodies (15 µg ml$^{-1}$, mouse, PharMingen, Hamburg) directed against extra- and intracytoplasmic domains (*DCC* antibodies and protein G/Sepharose were incubated for 4–6 h before). *DCC*-specific immunoprecipitates were recovered, washed, resuspended in Laemmli's sample buffer, and then subjected to SDS polyacrylamide gel electrophoresis (PAGE). The protein was transferred to a nitrocellulose membrane (Schleicher & Schuell, Dassel) by a semidry transblot system (Biometra, Göttingen). The DCC protein in the immunoprecipitates was detected by the ECL immunoblot assay (Amersham, Braunschweig) and subsequent exposure to Hyperfilm-βMax (Amersham).

### Immunohistochemical analysis of *DCC* expression

For immunohistochemistry, paraffin-embedded specimens of osteosarcoma were available from 13 patients. Immunohistochemistry (IHC) was performed using the alkaline–antialkaline phosphatase (APAAP) system as described previously (Cordell et al, 1984). Briefly, undecalcified 4-µm sections were dewaxed in xylene and rehydrated in descending concentrations of ethanol. For antigen retrieval of the *DCC* epitope, samples were boiled in a microwave oven (750 W) for 10 min in citrate buffer (0.1 M; pH 6.0). After extensive washing in Tris-buffered saline (145 mM sodium chloride, 20 mM Tris; pH 7.5) the sections were exposed to the primary antibody (mouse, monoclonal, 8 µg ml$^{-1}$, PharMingen, no. 15041 A). A secondary rabbit anti-mouse antibody (Dako, Hamburg) followed by the APAAP complex (Dako) was used for detection. Naphtol-AS-biphosphate (Sigma-Aldrich, Deisenhofen) and Neufuchsin (Merck, Darmstadt) were used for light microscopic visualization of the signal. Normal colon mucosa served as positive control, whereas for negative controls the primary antibody was omitted.

**Figure 2** *DCC* mRNA expression in osteosarcoma tumour specimens and tumour cell lines. (**A**) Biplex *DCC/G6PD* RT–PCR. *DCC* cDNA was amplified by PCR and analysed on 2% agarose gets. The *DCC*-specific fragment has a length of 233 bp. G6PD specific primers were used for control of amplification efficiency of weakly expressed genes. G6PD primers generated a product of 149 bp length. M, molecular size marker; lanes 1, 2: osteosarcoma cell lines TE85, Wo-OS; lanes 3–10: osteosarcoma specimens OS18, OS17, OS12, OS11, OS21, OS20, OS19, OS5; N, normal non-malignant osseous tissue. (**B**) Biplex *DCC*/β-actin RT–PCR. Agarose gel electrophoresis of generated *DCC* products (233 bp) and co-amplitied β-actin fragments (446 bp). Lanes 1–7, 11, 12: osteosarcoma specimens OS7, OS9, OS3, OS23, OS24, OS8, OS13, OS16, OS14. Lanes 8–10: colon carcinoma cell lines SW48, SW403, SW948. C, negative control (RT-PCR without RNA template). Using G6PD-specific primers an intense band was found in OS13, rendering the vast reduction of *DCC* expression a specific event. (**C**) Southern analysis of the PCR products presented in (**B**) using a 1.65-kb *DCC* cDNA and a β-actin-specific probe. The signal intensity of *DCC*-specific products was used for semiquantitation of *DCC* mRNA expression

### Flow-cytometric analysis of *DCC* expression

An indirect immunofluorescent technique was performed using a Becton Dickinson FACS scan as described previously (Pollice et al, 1992). The specific antibody (mouse, monoclonal, PharMingen) was again directed against the intracytoplasmic domain of the DCC protein.

© Cancer Research Campaign 1997

**Table 3** *DCC* expression analysis

| Specimen | Relative abundance of *DCC* transcripts | RT-PCR AUC (A) | Immunoblot | IHC/ flowcytometry |
|---|---|---|---|---|
| Normal osteoblast | +++ | 8.3 (±0.78 s.d.) | Negative | +++ |
| OS1 | ++++ | 10.0 | Negative | +++ |
| OS2 | ++++ | 9.5 | Negative | +++ |
| OS3 | +++ | 8.3 | Negative | +++ |
| OS4 | +++ | 8.9 | Negative | +++ |
| OS5 | +++ | 7.8 | ND | ND |
| OS6 | ++ | 5.2 | ND | ND |
| OS7 | + | 4.6 | ND | ND |
| OS8 | ++ | 5.2 | Negative | ND |
| OS9 | + | 2.3 | ND | (+) |
| OS10 | + | 3.8 | ND | –[a] |
| OS11 | + | 3.5 | ND | ND |
| OS12 | (–) | 0.9 | ND | – |
| OS13 | – | 0 | ND | – |
| OS14 | (–) | 0.9 | ND | ND |
| OS15 | (–) | 0.3 | ND | – |
| OS16 | (–) | 0.3 | ND | – |
| OS17 | (–) | 0.4 | Negative | – |
| OS18 | (–) | 1.1 | ND | (+) |
| OS19 | (–) | 0.8 | ND | ND |
| OS20 | +++ | 7.2 | ND | ND |
| OS21 | (–) | 0.7 | ND | ND |
| OS22 | + | 2.6 | ND | ND |
| OS23 | +++ | 7.5 | ND | ND |
| OS24 | + | 4.7 | Negative | ++ |
| OS25 | ++ | 6.9 | Negative | ++ |
| Cell lines | | | | |
| TE85 | – | 0 | Negative | –[b] |
| Saos-2 | – | 0 | Negative | –[b] |
| KHOS-240S | – | 0 | Negative | –[b] |
| U-2 OS | (+) | 2.1 | Negative | –[b] |
| Wo-OS | – | 0 | ND | ND |
| RD-ES | ++ | 5.8 | Positive | +++[b] |
| A-204 | – | 0 | Negative | ND |
| CCRF-CEM | – | 0 | Negative | –[b] |
| SW48 | + | 4.6 | ND | ND |
| SW403 | – | 0 | ND | ND |
| SW948 | (–) | 0.8 | ND | ND |
| SW1116 | (+) | 2.2 | ND | ND |
| HCT116 | (+) | 1.6 | ND | ND |
| SW1463 | ++ | 6.1 | ND | ND |

Normal osteoblast: normal bone tissue devoid of bone marrow cells was used for RT–PCR and immunoblot analysis; *DCC* expression of normally differentiated osteoblasts was confirmed by immunohistochemistry. OS, osteogenic sarcoma. Origins of cell lines are listed in Table 2 The relative abundance of *DCC*-specific transcripts was determined by a semiquantitative RT–PCR-based assay. After Southern transfer, *DCC* PCR products were hybridized with a $^{32}$P-CTP-labelled *DCC* cDNA. The intensity of the autoradiographic signals, which reflect the relative amounts of *DCC* transcripts, was measured densitometrically as the area under the curve of absorption AUC (A). AUCs were compared with AUC (A) values based on *DCC* PCR data from serially diluted cDNAs of normal osseous tissue specimens (Figure 1). Semiquantitation of AUCs (A): ++++, *DCC* expression > normal osseous tissue; +++, 1:1–1:10 dilution of normal osteoblast cDNA; ++, > 1:10 ≤ 1:20 dilution; +, > 1:20 ≤1:50; (+), > 1:50≤1:100; (–), >1:100 dilution; – *DCC* expression detectable. IHC, immunohistochemistry: –, negative; (+), very faint, +, weak, ++, moderate; +++, strong IHC staining intensity for monoclonal anti-*DCC* antibody directed against the intracytoplasmatic domain. [a] Negative staining of transformed osteoblasts, faint *DCC* positivity of granulation tissue cells. [b] FACS scan flowcytometric analysis only; s.d., standard deviation; ND, not determined.

### RESULTS

### *DCC* mRNA expression

Northern analysis was largely unsuccessful in demonstrating any *DCC* transcript in normal as well as malignant osseous tissue. Therefore, a sensitive RT-PCR-based approach was chosen to amplify DCC transcripts from cDNA to evaluate the level of *DCC* gene expression. In normal osseous tissue, *DCC* transcription was consistently detected with a low variability between different individuals. Autoradiographic signal intensities of PCR products from serially diluted cDNAs from normal osseous specimens served as semiquantitative reference values for the degree of DCC transcription (Figure 1).

The analysis of DCC specific RT-PCR products by gel electrophoresis is demonstrated in Figure 2A and B), which presents a selection of osteosarcomas and tumour cell lines. As expected, the

© Cancer Research Campaign 1997

B Arithmetic/Linear

**Figure 3** (**A**) DCC protein expression in various tumour cell lines, *DCC* transcribing osteosarcomas and normal osseous tissue. Lysates [approximately 100 µg total protein or *high molecular weight fractionated protein (> 100 kDa with the use of Centrikon filters)] were studied by SDS-PAGE on 8% gels and an ECL immunoblot analysis. The DCC protein is expected to migrate in a rather broad region with a molecular weight of approximately 180 000–210 000. Lanes 1, 9, 10: Ewing's sarcoma cell line RD-ES; lanes 2–5: T-ALL cell line CCRF-CEM, osteosarcoma cell lines U2-OS, SaOS-2, TE85; lanes 6, 7: osteosarcoma specimens OS25, OS1; lane 8: normal osseous tissue. The faint signals in lanes 6–8, which co-migrate with the DCC protein, are considered non-specific. (**B**) FACS scan flowcytometric analysis of DCC protein expression in Ewing's sarcoma cell line RD-ES, T-ALL cell line CEM, and osteosarcoma cell lines TE85, SAOS, U2OS, KHOS. With the use of a monoclonal antibody directed against the cytoplasmic domain of the DCC protein the Ewing's sarcoma derived cell line RD-ES exhibited a positive signal indicated by a non-confluent peak

*© Cancer Research Campaign 1997*

**Figure 4** Immunohistochemical analysis of *DCC* in normally differentiated and transformed osteoblasts. (**A**) Myositis ossificans. The centripetal osteoblastic differentiation is linked to an increase in DCC protein expression from undifferentiated mesenchymal cells to mature non-transformed osteoblasts. (**B**) The photomicrograph of a human high-grade osteosarcoma (OS 18; predominantly chondroblastic), which revealed a vast reduction in *DCC* mRNA expression demonstrates a very faint *DCC* signal in a few tumour cells. (**C**) A strong cytoplasmatic *DCC* signal was detected in an osteoblastic high-grade osteosarcoma (OS 4) with large amounts of *DCC* transcripts. (**D**) DCC protein expression in a low-grade osteosarcoma revealing reduced amounts of *DCC* transcripts (OS24). The DCC signal is detected as a red staining predominantly within the cytoplasm of cells (magnification 120×, counterstaining with haematoxylin)

Southern blot analyses of *DCC* PCR products (Figure 2C) that were used for semiquantitation proved more sensitive than the in-gel measurement of PCR fragments (Figure 2B). Table 3 gives an overview of *DCC* RT-PCR data from normal osseous tissue, tumour specimens and cell lines. Eight out of 19 high-grade osteosarcomas (six primary tumours, two lung metastases; OS12–19) had a nearly total extinction of *DCC* expression with a relative abundance of *DCC* transcripts of less than 1% of normal osseous tissue cells as estimated from the cDNA dilution graph delineated in Figure 1. In another six high-grade osteosarcomas (four primary tumours, one metastasis each in lung and spine; OS6–11) the *DCC* transcription was found to be reduced to less than 5% of normal. A normal or even increased *DCC* expression was found in the remaining five high-grade osteosarcomas comprising three primary tumours and two lung metastases (OS1-5). Among the three periosteal intermediate-grade osteosarcomas, two tumours were found to have decreased or almost undetectable *DCC* expression (OS21, 22). Two out of three low-grade osteosarcoma specimens (OS24, 25) revealed reduced levels of *DCC* to varying degrees, whereas OS23 was found to express *DCC* transcripts within the range of normal bone tissue.

In three cases, two different tumour specimens from one patient each were analysed by RT-PCR. The lung metastases of patient no. 5 almost completely lost the *DCC* expression within 1 year of progressive disease (OS5, 19). In patient no. 19 the primary intermediate-grade osteosarcoma initially exhibited a strong DCC expression, which was lost when the tumour recurred 4 months later (OS20, 21; Figure 2A). The low-grade osteosarcoma OS24 represents a second local relapse with a rather low level of *DCC* expression which was found to be somewhat higher in its concomitant lung metastasis specimen (OS25).

Most of the cell lines investigated in this study demonstrated very low or even absent *DCC* expression, that is TE 85, Wo-OS, Saos-2, U2-OS, A-204, SW 403, SW 948, SW 1116, HCT 116 and CCRF-CEM. The colon carcinoma cell lines SW1463 and SW48, as well as the Ewing's sarcoma cell line RD-ES, showed easily detectable *DCC* transcripts but at a lower level than that found in normal osteoblasts.

### DCC protein expression

The initial studies of endogenous DCC protein expression were performed on a variety of cell lines (TE 85, Saos-2, U2-OS,

© Cancer Research Campaign 1997

KHOS-24OS, RD-ES, CCRF-CEM) using a combined immunoprecipitation and immunoblotting assay as well as flow-cytometric technique. A series of various monoclonal antibodies were tested that were directed against extra- or intracytoplasmic domains of the DCC protein. The most reliable results were obtained with use of a monoclonal antibody vs the intracytoplasmatic domain (PharminGen). Only the human Ewing's sarcoma cell line RD-ES demonstrated an approximately $M_r$ 180 000 protein that was detected both by the immunoblot assay and the flow cytometric technique (Figure 3A and B). Using the combined immunoprecipitation and immunoblot assay, DCC protein expression was not detectable in a variety of normal osseous tissues or osteosarcomas revealing high levels of *DCC* transcription (Figure 3A). Immunohistochemical analysis, however, proved to be more sensitive than the immunoblot approach. The DCC protein could be clearly demonstrated in normal osteoblasts/osteocytes (Figure 4A). Moreover, in a subset of 14 available osteosarcomas (see Table 3) DCC protein expression correlated well with the level of *DCC* transcription based on an RT-PCR approach (Figure 4B–D). In normal tissues, the most pronounced staining for *DCC* was found in mature osteocytes/osteoblasts. A less intensive *DCC* immunoreactivity was observed in immature mesenchymal cells presumably differentiating to osteoblastic cells (Figure 4A). Additionally, a faint to moderately intense staining was found in macrophages, fibroblasts and sometimes in endothelial cells as cellular components of granulation tissues (data not shown).

### *DCC* rearrangement analysis

To determine whether the abnormalities of *DCC* expression could be related to any gross structural rearrangements we performed Southern blot analyses of *Eco*RI-restricted genomic DNA using the *1.65* DCC cDNA as described above. Originally, this probe was the longest clone that was isolated during the construction of a *DCC* cDNA library from RNA of a colon carcinoma cell line (Fearon et al, 1990). Gross alterations within the *DCC* gene were detected in only two colon carcinoma cell lines (SW 1116, HCT 116) expressing reduced amounts of *DCC* transcripts, but not in osteosarcomas and the remaining colon carcinoma cell lines, which revealed a constitutional hybridization pattern of *Eco*RI-restricted genomic DNA (data not shown).

### DISCUSSION

In summary, the present study demonstrates a reduction or even a loss of *DCC* gene expression in the majority of human high-grade osteosarcomas and osteosarcoma cell line derivates. This conclusion could be drawn from *DCC* RNA as well as protein expression analyses. In view of the putative tumour-suppressor characteristics of the *DCC* gene it is tempting to assume that its reduction or loss of expression in many high-grade osteosarcomas may be important in the pathogenesis of this tumour entity. As mentioned before a loss of heterozygosity within the *DCC* locus has been frequently observed in other malignancies, and expression studies revealed a remarkable relationship between *DCC* expression, cellular differentiation and tumorigenesis. A further line of evidence that the *DCC* gene may be a tumour suppressor resulted from functional *DCC* transfection studies. The introduction of a full-length *DCC* cDNA into nitrosomethylurea-transformed human epithelial cells resulted in a suppression of tumorigenicity (Klingelhutz et al, 1994).

In colon carcinoma, a loss of *DCC* expression can be considered a late event in tumour progression (Kikuchi-Yanoshita et al, 1992). Moreover, it has been suggested that *DCC* may act as a metastatic suppressor in colon carcinoma (Ookawa et al, 1993). We found a frequent loss or decrease of *DCC* expression not only in metastatic but also in primary disease, possibly indicating an early metastatic potential of high-grade osteosarcoma. In fact, 10–20% of patients have detectable metastases at primary diagnosis, and it has been assumed that up to 80% of osteosarcomas may have spread occult micrometastases at diagnosis (Link and Eilber, 1993). In some cases, however, full transcription of *DCC* has been found in metastatic osteosarcoma cells, indicating that its loss of function is not a necessary premise for a distant spread postulating alternative pathways of the metastatic process. The analysis of *DCC* expression in follow-up specimens of two cases of osteosarcoma (OS5, 19 and OS20, 21) suggests, at least in these tumours, an inverse relationship between the magnitude of *DCC* transcription and progressive disease. It is noteworthy that *DCC* expression of transformed osteoblasts does not depend on the production of any specific type of extracellular matrix, i.e. *DCC* status correlates rather with the grading of a given tumour than with its histological subtype.

In osseous tissue the immunoblot approach to *DCC* expression was largely unsuccessful, confirming earlier studies on DCC protein expression in various other tissues (Hedrick et al, 1994; Reale et al, 1994). Only the Ewing's sarcoma cell line RD-ES produced a clear signal at about 180 000 kDa in the immunoblot analysis. The transmembrane localization of the DCC protein may have rendered immunohistochemistry more suitable for demonstrating its expression than techniques relying on whole-tissue homogenates. There is an obvious discrepancy between RD-ES culture cells and cells from native osseous tissues with regard to *DCC* expression in the RT–PCR assay compared with the immunoblot technique. Using the RT–PCR assay both cell types demonstrated easily detectable abundances of *DCC* transcripts. By contrast, immunoblotting combined with immunoprecipitation revealed *DCC* expression only in the RD-ES cells. The lack of detectable DCC protein in osseous tissue cells might result from degradation of the protein product during sample preparation and processing that appears to be more relevant to protease-containing tissue homogenates than to culture cells.

Of the human tumour cell lines studied, only one colon carcinoma and the Ewing's sarcoma cell line RD-ES revealed easily detectable amounts of *DCC* transcripts, whereas most others were found to have a substantial decrease in *DCC* expression including the osteosarcoma-derived cell lines. Results from three colon carcinoma cell lines differ from data obtained in earlier studies with regard to the extent of *DCC* expression (Fearon et al, 1990). These differences may be attributable to an inconsistent mutational inactivation of the *DCC* gene or aberrant alternative *DCC* mRNA splicing processes, which were described previously (Reale et al, 1994). A mere tissue culture artefact from the in vitro growth of cells seems to be unlikely, as we identified several cell lines with a consistent *DCC* transcription. Intriguingly, we found comparably moderate to high abundances of *DCC* transcripts in each of eight investigated Ewing's sarcomas (data not shown) and the derivative cell line RD-ES, suggesting that *DCC* is not likely to undergo gene silencing under in vitro conditions.

In most human malignancies, suppressor genes appear to be inactivated at the DNA level. Numerous molecular mechanisms of gene inactivation have been described, including point mutations, chromosomal deletions, rearrangements and insertions. Using a

© Cancer Research Campaign 1997

1.65-kb *DCC* cDNA probe we were not able to demonstrate any rearrangement or deletion in *Eco*RI-restricted genomic DNA from osteosarcoma. This probe, however, does not fully constitute the open reading frame of 4341 bp of the *DCC* gene, and thus neither rearrangements outside this region nor subtle alterations within would be detected. More comprehensive studies using a set of polymorphic 18q probes and mapping strategies in addition to an exhaustive mutational analysis of the 1.4 Mb *DCC* gene may be necessary to search for specific DNA alterations leading to a decreased *DCC* transcription in human osteosarcoma. In view of the enormous size of the *DCC* gene, mutational analysis is probably the most laborious way to look for alterations that may specifically inactivate *DCC* as a tumour-suppressor gene at DNA level. The hitherto evaluated portions of the *DCC* sequence compose less than 1% of the gene (Cho et al, 1994; E Fearon, 1996, personal communication). Furthermore, studies on *DCC* RNA processing should complement our approach to the *DCC* gene in osteosarcoma, as aberrant alternative splicing of *DCC* mRNA appears to play a role in other malignancies (Reale et al, 1994; Ekstrand et al, 1995). Finally, the functional restoration of the *DCC* gene in *DCC*-deficient osteosarcoma cells will be of great interest in order to provide further evidence that *DCC* serves as a tumour-suppressor gene in human osteosarcoma and that the loss or decrease of its expression is more than an epigenetic phenomenon.

### ACKNOWLEDGEMENTS

We are grateful to Drs Michael Reale and Eric Fearon for advice. Furthermore, we would like to thank Dr Bert Vogelstein for providing the 1.65 *DCC* cDNA probe and Belinda Weber for excellent technical assistance. This study was supported in part by the Foerdergemeinschaft Kinderkrebshilfe e. V. Hamburg and Hamburger Krebsgesellschaft. BA was supported by a grant from the Kind-Philipp-Stiftung.

### REFERENCES

Adams MD, Dubnick M, Kerlavage AR, Moreno R, Kelley JM, Utterback TR, Nagle JW, Fields C and Venter Craig (1992) Sequence identification of 2,375 human brain genes. *Nature* **355**: 632–634

Chirgwin JM, Przybyla AE, MacDonald RJ and Rutter WJ (1979) Isolation of biologically active ribonucleic acid from sources enriched in ribonucleases. *Biochemistry* **24**: 5294–5299

Cho KR, Oliner JD, Simons JW, Hedrick L, Fearon ER, Preisinger AC, Hedge P, Silverman GA and Vogelstein B (1994) The DCC gene: structural analysis and mutations in colorectal carcinomas. *Genomics* **19**: 525–531

Chomczynski P and Sacchi N (1987) Single-step method of RNA isolation by acid guanidinium thiocyanate–phenol–chloroform extraction. *Anal Biochem* **162**: 156–159

Cordell JL, Falini B, Erber WN, Gosh AL, Abdulaziz Z, Mac Donald S, Pulford KAF, Stein H and Mason DJ (1984) Immunoenzymatic labeling of monoclonal antibodies using immune complexes of alkaline phosphatase and monoclonal antialkaline phosphatase (APAAP complexes). *J Histochem Cytochem* **32**: 219–229

Cropp CS, Lidereau R, Campbell G, Champene MH and Callahan R (1990) Loss of heterozygosity on chromosomes 17 and 18 in breast carcinoma: two additional regions identified. *Proc Natl Acad Sci USA* **87**: 7737–7741

Ekstrand BC, Mansfield TA, Bigner SH and Fearon ER (1995) DCC expression is altered by multiple mechanisms in brain tumours. *Oncogene* **11**: 2393–2402

Fearon E, Cho KR, Nigro J, Kern S, Simons J, Ruppert JM, Hamilton S, Preisinger A, Thomas G, Kinzler K and Vogelstein B (1990) Identification of a chromosome 18q gene that is altered in colorectal cancers. *Science* **247**: 49–56

Friend S, Bernards R, Rogelj S, Weinberg RA, Rapaport JM, Albert DM and Dryja TP (1986) A human DNA segment with properties of the gene that predisposes to retinoblastoma and osteosarcoma. *Nature* **323**: 643–646

Gao X, Honn KV, Grignon D, Sakr W and Chen YQ (1993) Frequent loss of expression and loss of heterozygosity of the putative tumor suppressor gene DCC in prostatic carcinomas. Cancer Res **53**: 2723–2727

Hedrick L, Cho KR, Fearon ER, Wu T-C, Kinzler KW and Vogelstein B (1994) The DCC gene product in cellular differentiation and colorectal tumorigenesis. *Genes* Dev **8**: 1174–1183

Hohne MW, Halatsch M-E, Kahl GF and Weinel RJ (1992) Frequent loss of expression of the potential tumor suppressor gene DCC in ductal pancreatic adenocarcinoma. Cancer Res **52**: 2616–2619

Kikuchi-Yanoshita R, Konishi M, Fukunari H, Tanaka K and Miyaki M (1992) Loss of expression of the DCC gene during progression of colorectal carcinomas in familial adenomatous polyposis and non-familial adenomatous polyposis patients. *Cancer Res* **52**: 3801–3803

Klingelhutz AJ, Hedrick L, Cho KR and McDougall JK (1995) The DCC gene suppresses the malignant phenotype of transformed human epithelial cells. *Oncogene* **10**: 1581–1586

Link M and Eilber F (1993) Osteosarcoma. In *Principles and Practice of Pediatric Oncology*. Pizzo P and Poplack D (eds), pp. 841–866. Lippincott: Philadelphia.

Maniatis T, Fritsch E and Sambrook J (1989) *Molecular Cloning: a Laboratory Manual*. Cold Spring Harbor Laboratory Press: Cold Spring Harbor

Miller CW, Aslo A, Tsay C, Slamon D, Ishizaki, K, Toguchida J, Yamamuro T, Lampkin B and Koeffler HP (1990) Frequency and structure of p53 rearrangements in human osteosarcoma. *Cancer Res* **50**: 7950–7954

Miyake K, Inokuchi K, Dan K and Nomura T (1993) Expression of the DCC gene in myelodysplastic syndromes and overt leukemia. *Leukemia Res* **17**: 785–788

Mulligan LM, Matlashewski GJ, Scrable HJ and Cavenee WK (1990) Mechanisms of p53 loss in human sarcomas. *Proc Natl Acad Sci USA* **87**: 5863–5867

Nakajima-Iijima S, Hamada H, Reddy P, Takanuga T (1985) Molecular structure of the human cytoplasmic β-actin gene: intraspecies homology of sequences in the introns. *Proc Natl Acad Sci USA* **82**: 6133–6137

Narayanan R, Lawlor KG, Schaapveld RQJ, Cho KR, Vogelstein B, Tran PB-V, Osborne MP and Telang NT (1992) Antisense RNA to the putative tumor-suppressor gene DCC transforms Rat-1 fibroblasts. *Oncogene* **7**: 553–561

Ookawa K, Sakamoto M, Hirohashi S, Yoshida Y, Sugimura T, Terada M and Yokota J (1993) Concordant p53 and DCC alterations and allelic losses on chromosomes 13q and 14q associated with liver metastases of colorectal carcinoma. *Int J Cancer* **53**: 382–387

Persico MG, Toniolo D, Nobile C, D'Urso M and Luzzatto L (1981) cDNA sequences of human glucose 6-phosphate dehydrogenase cloned in pBR322. *Nature* **294**: 778–780

Pollice AA, McCoy JP, Jr., Shackney SE, Smith CA, Agarwal J, Burholt DR, Janocko LE, Hornicek FJ, Singh SG and Hartsock RJ (1992) Sequential paraformaldehyde and methanol fixation for simultaneous flow cytometric analysis of DNA cell surface proteins and intracellular proteins. *Cytometry* **13**: 432–444

Porfiri E, Secker-Walker LM, Hoffbrand AV and Hancock JF (1993) DCC tumor supressor gene is inactivated in hematologic malignancies showing monosomy 18. *Blood* **81**: 2696–2701

Reale M, Hu G, Zafar AI, Getzenberg RH, Levine SM and Fearon ER (1994) Expression and alternative splicing of the deleted in colorectal cancer (DCC) gene in normal and malignant tissues. *Cancer Res* **54**: 4493–4501

Scheck AC and Coons SW (1993) Expression of the tumor suppressor gene DCC in human gliomas. *Cancer Res* **53**: 5605–5609

Thompson AM, Morris RG, Wallace M, Wyllie AH, Steel CM and Cartel DC (1993) Allele loss from 5q21 (APC/MCC) and 18q21 (DCC) and DCC mRNA expression in breast cancer. *Br J Cancer* **68**: 64–68

Toguchida J, Ishizaki K, Sasaki MS, Nakamura Y, Ikenaga M, Kato M, Sugimot M, Kotoura Y and Yamamuro T (1989) Preferential mutation of paternally derived RB gene as the initial event in sporadic osteosarcoma. *Nature* **338**: 156–158

Uchino S, Tsuda H, Noguchi M, Yokota J, Terada M, Saito T, Kobayashi M, Sugimura T and Hirohashi S (1992) Frequent loss of heterozygosity at the DCC locus in gastric cancer. *Cancer Res* **52**: 3099–3102

Winkler K, Beron G, Delling G, Heise U, Kabisch H, Purfurst C, Berger J, Ritter J, Jürgens H, Gerein V, Graf N, Russe W, Gruemayer ER, Ertelt W, Kotz R, Preusser P, Prindull G, Brandeis W and Landbeck G (1988) Neoadjuvant chemotherapy of osteosarcoma: results of a randomized cooperative trial (COSS-82) with salvage chemotherapy based on histological tumor response. J Clin Oncol **6**: 329–337

Yamaguchi T, Toguchida J, Yamamuro T, Kotoura Y, Takada N, Kawaguchi N, Kaneko Y, Nakamura Y, Sasaki MS and Ishizaki K (1992) Allelotype analysis in osteosarcomas: frequent allele loss on 3q, 13q, 17p, and 18q. *Cancer Res* **52**: 2419–2424

© Cancer Research Campaign 1997


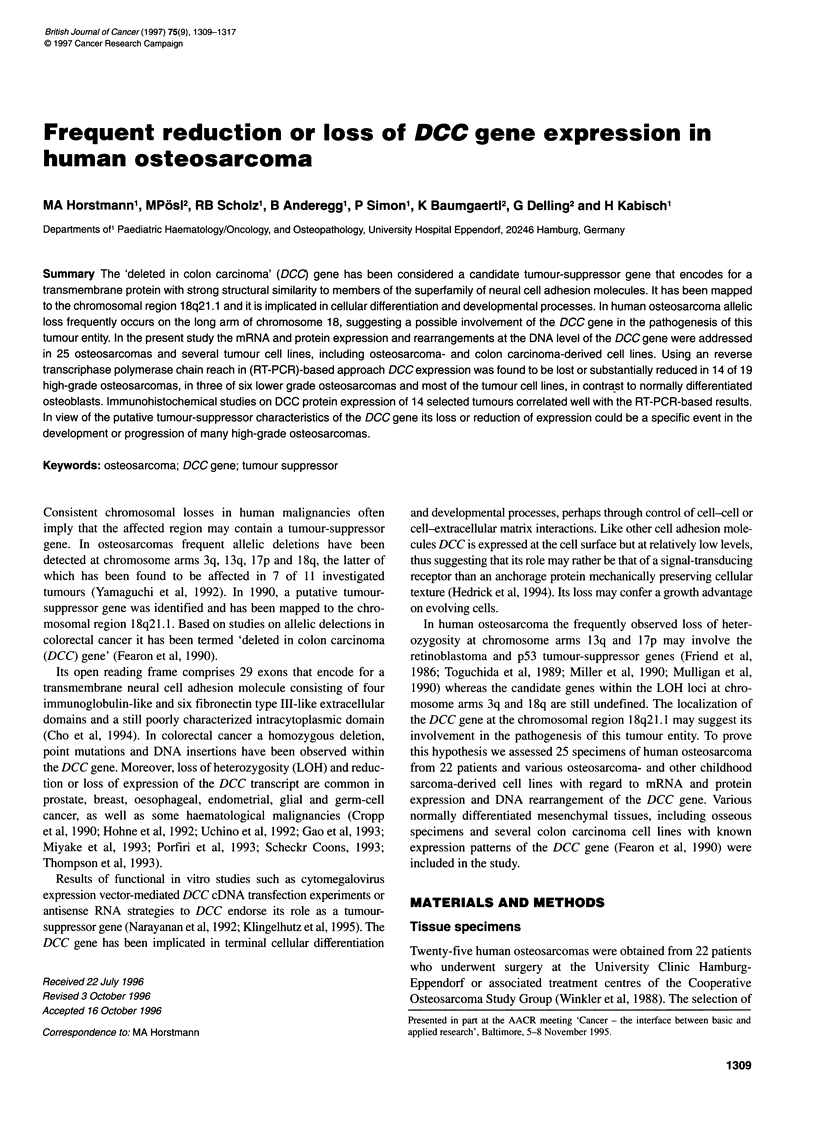

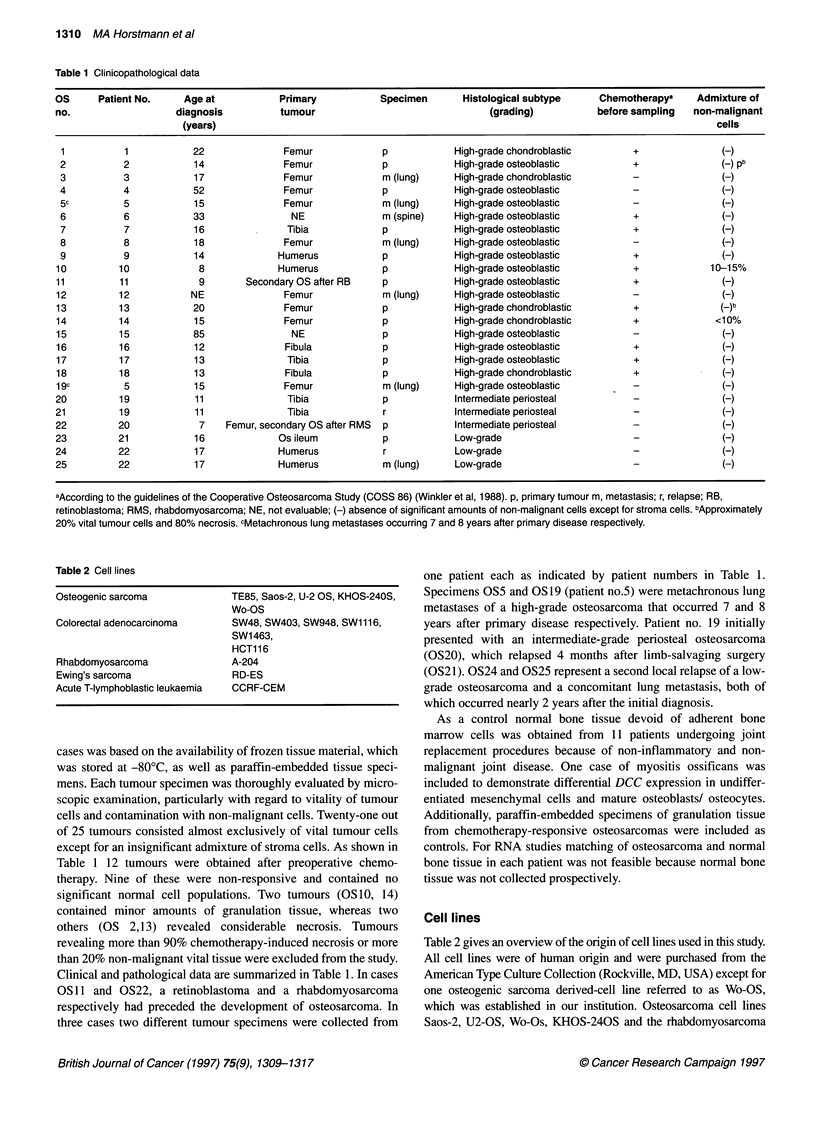

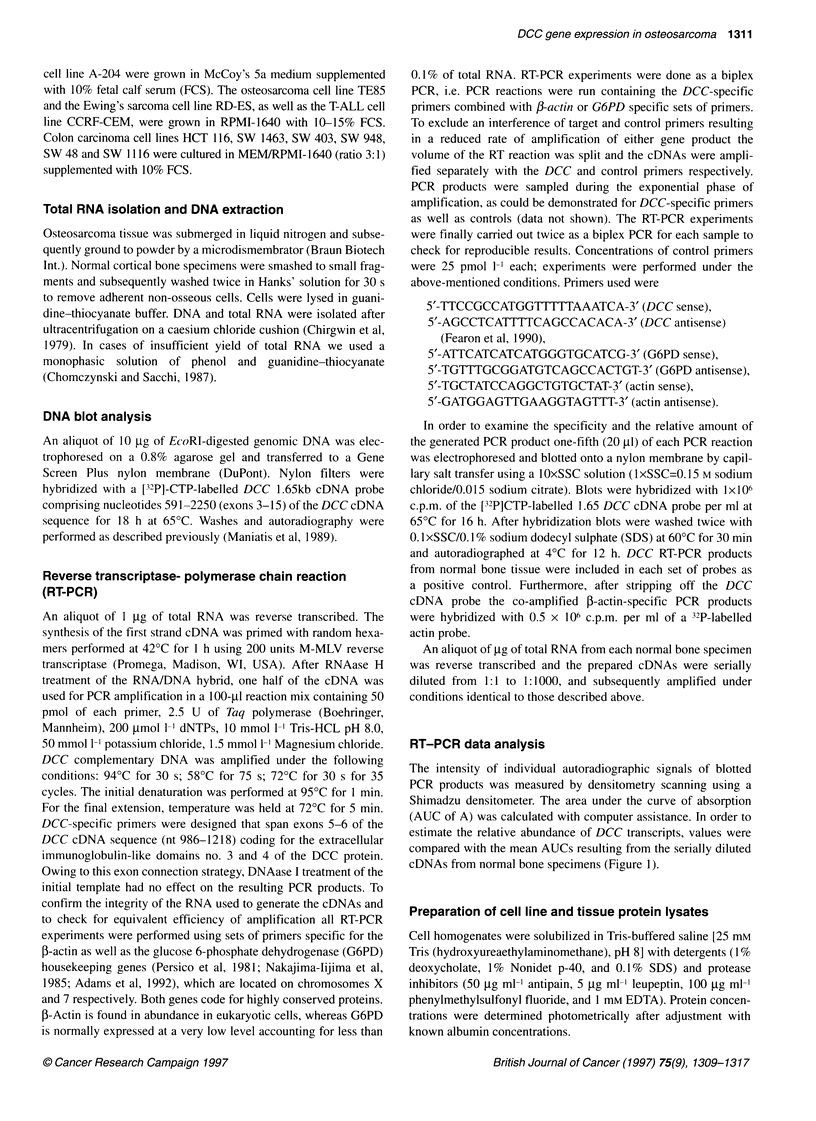

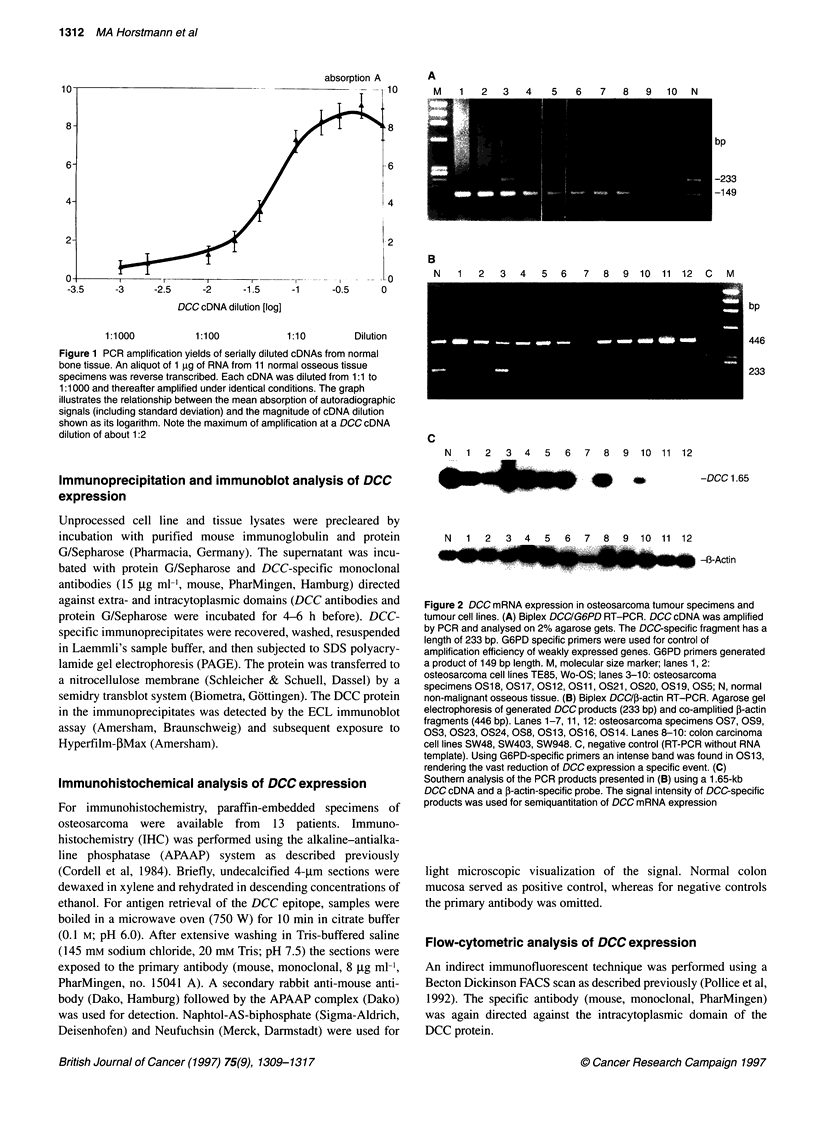

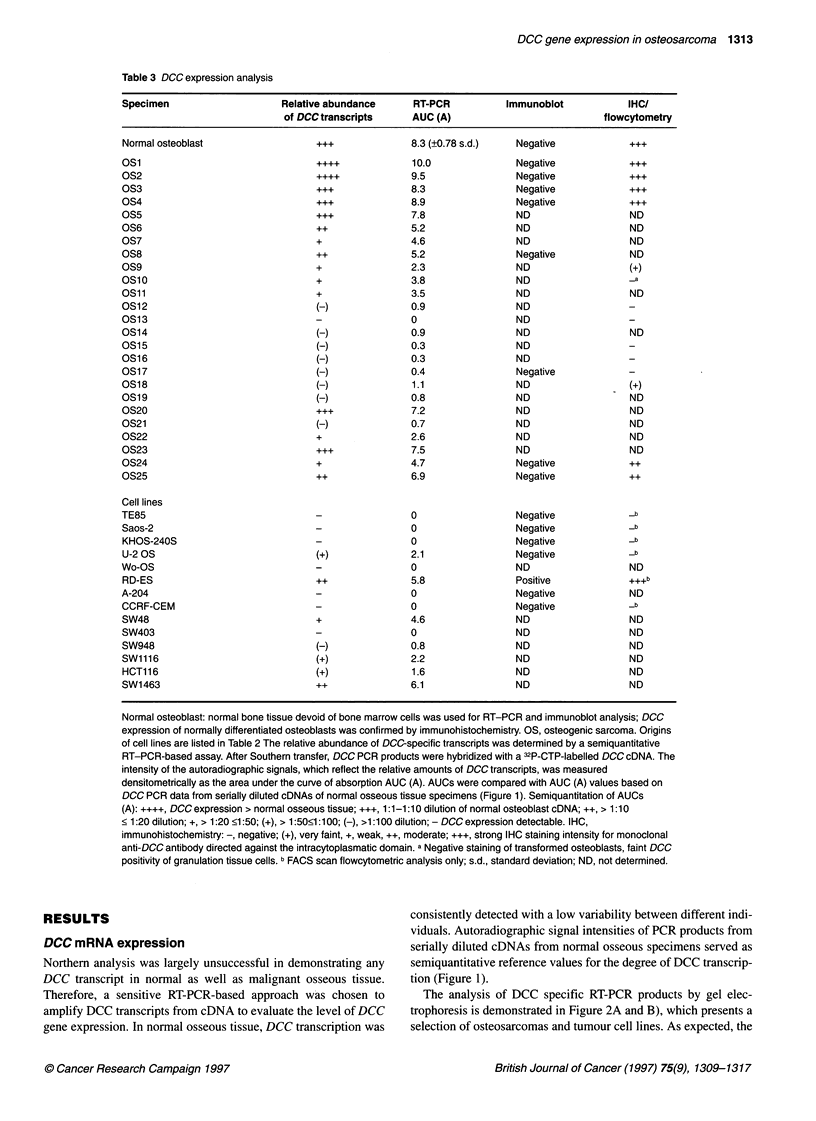

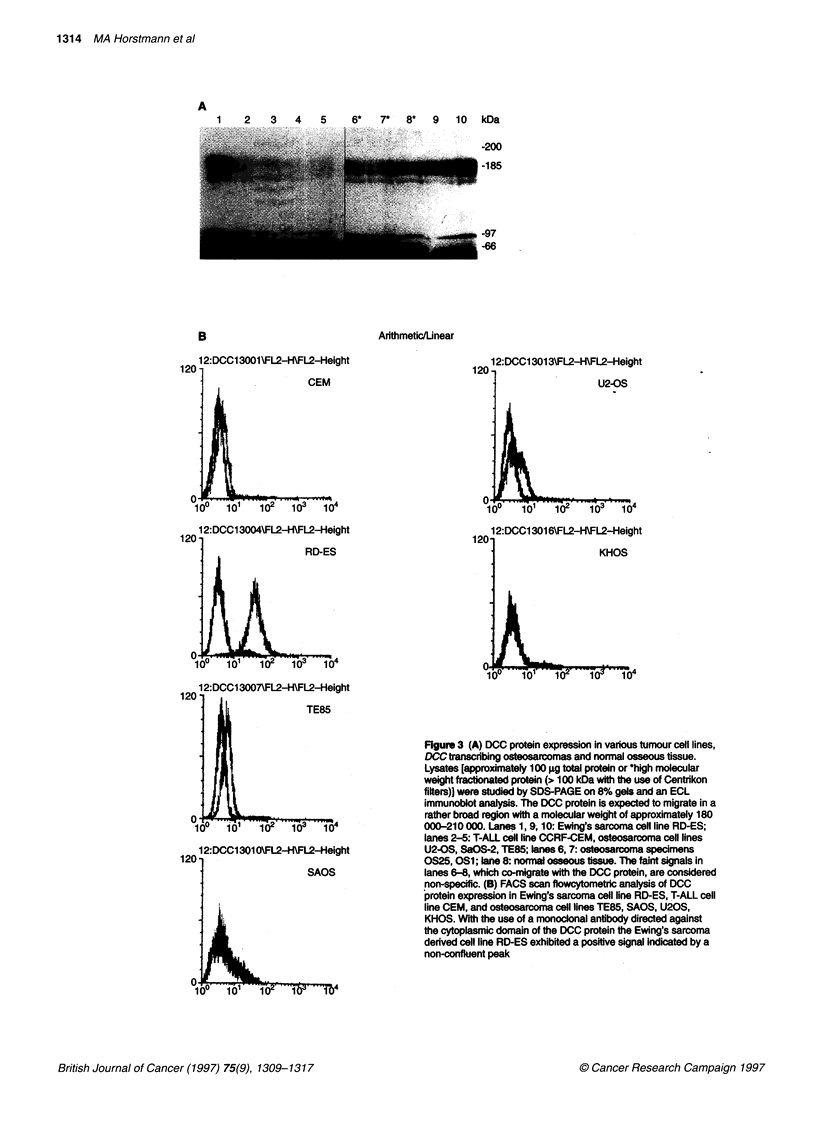

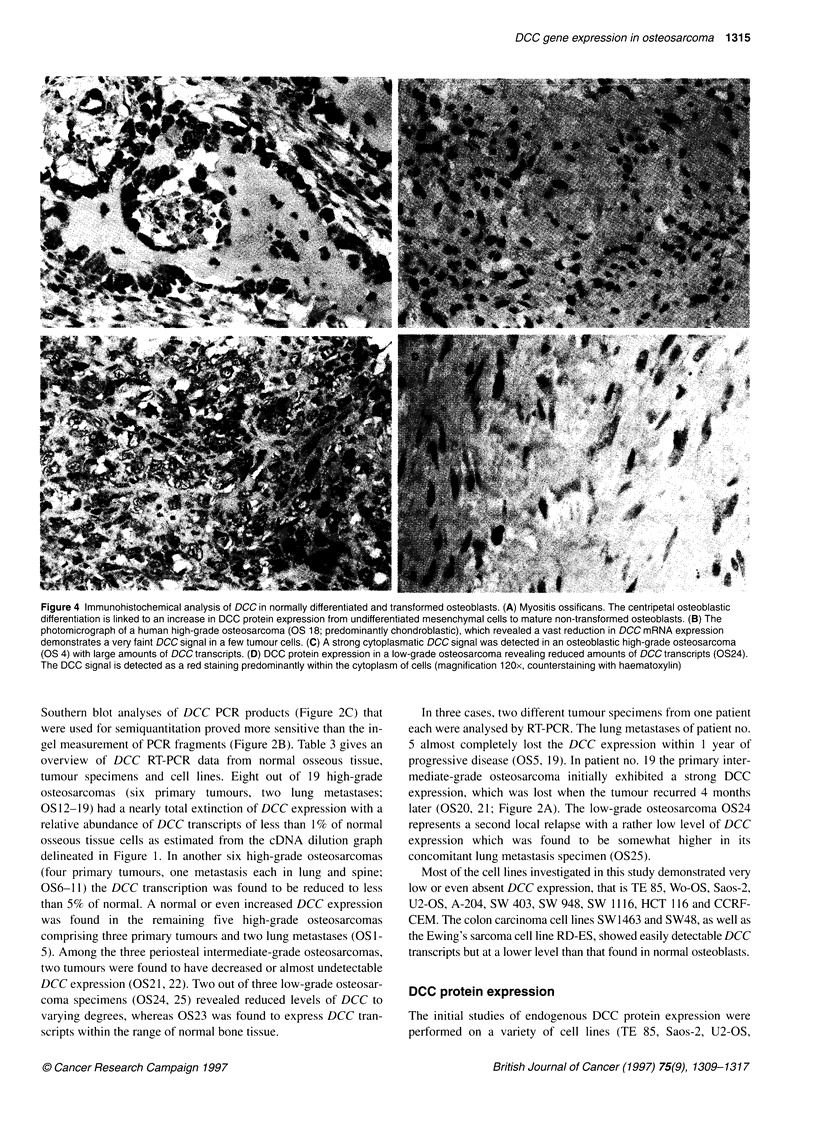

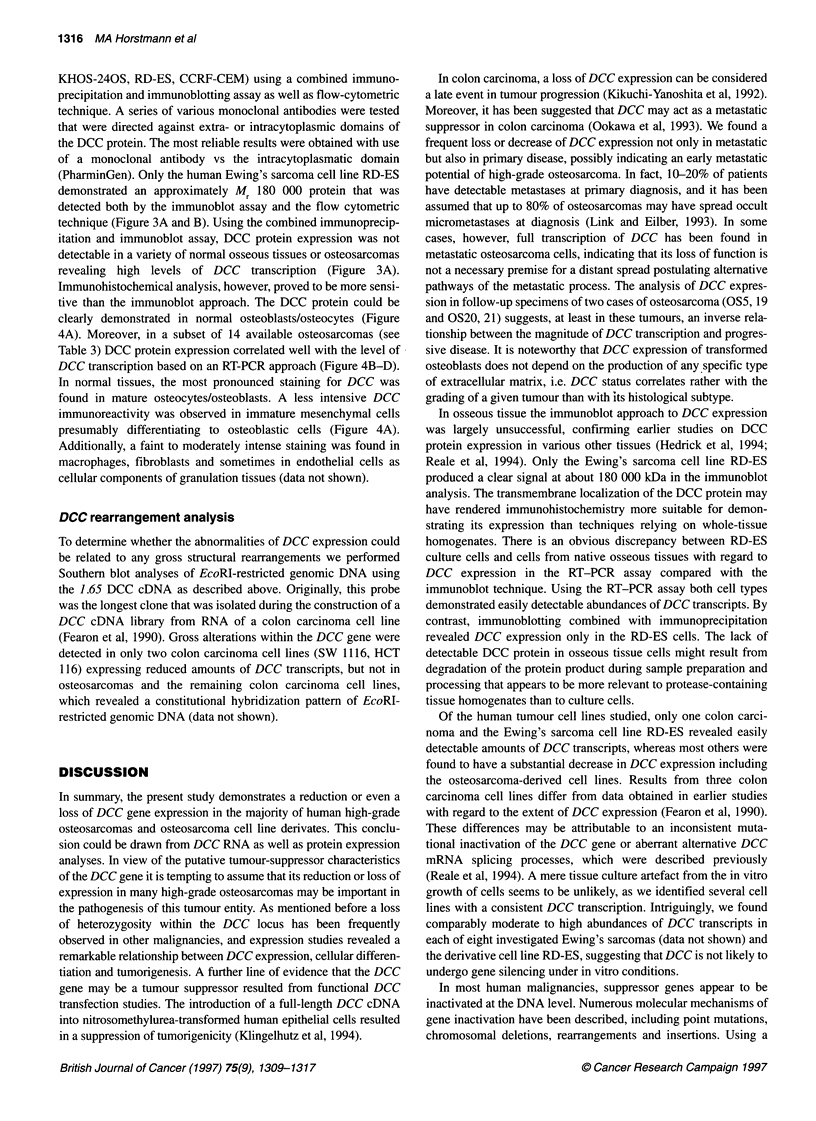

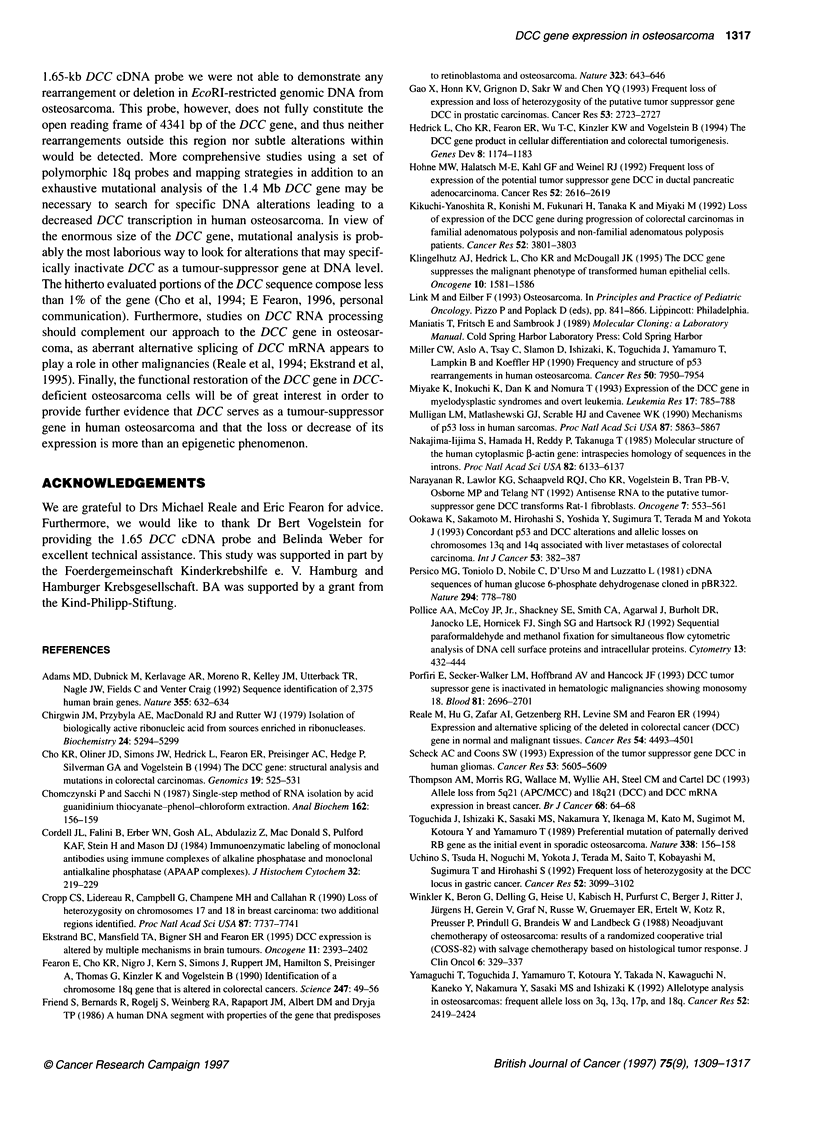


## References

[OCR_01100] Adams M. D., Dubnick M., Kerlavage A. R., Moreno R., Kelley J. M., Utterback T. R., Nagle J. W., Fields C., Venter J. C. (1992). Sequence identification of 2,375 human brain genes.. Nature.

[OCR_01105] Chirgwin J. M., Przybyla A. E., MacDonald R. J., Rutter W. J. (1979). Isolation of biologically active ribonucleic acid from sources enriched in ribonuclease.. Biochemistry.

[OCR_01110] Cho K. R., Oliner J. D., Simons J. W., Hedrick L., Fearon E. R., Preisinger A. C., Hedge P., Silverman G. A., Vogelstein B. (1994). The DCC gene: structural analysis and mutations in colorectal carcinomas.. Genomics.

[OCR_01115] Chomczynski P., Sacchi N. (1987). Single-step method of RNA isolation by acid guanidinium thiocyanate-phenol-chloroform extraction.. Anal Biochem.

[OCR_01120] Cordell J. L., Falini B., Erber W. N., Ghosh A. K., Abdulaziz Z., MacDonald S., Pulford K. A., Stein H., Mason D. Y. (1984). Immunoenzymatic labeling of monoclonal antibodies using immune complexes of alkaline phosphatase and monoclonal anti-alkaline phosphatase (APAAP complexes).. J Histochem Cytochem.

[OCR_01127] Cropp C. S., Lidereau R., Campbell G., Champene M. H., Callahan R. (1990). Loss of heterozygosity on chromosomes 17 and 18 in breast carcinoma: two additional regions identified.. Proc Natl Acad Sci U S A.

[OCR_01132] Ekstrand B. C., Mansfield T. A., Bigner S. H., Fearon E. R. (1995). DCC expression is altered by multiple mechanisms in brain tumours.. Oncogene.

[OCR_01136] Fearon E. R., Cho K. R., Nigro J. M., Kern S. E., Simons J. W., Ruppert J. M., Hamilton S. R., Preisinger A. C., Thomas G., Kinzler K. W. (1990). Identification of a chromosome 18q gene that is altered in colorectal cancers.. Science.

[OCR_01147] Gao X., Honn K. V., Grignon D., Sakr W., Chen Y. Q. (1993). Frequent loss of expression and loss of heterozygosity of the putative tumor suppressor gene DCC in prostatic carcinomas.. Cancer Res.

[OCR_01152] Hedrick L., Cho K. R., Fearon E. R., Wu T. C., Kinzler K. W., Vogelstein B. (1994). The DCC gene product in cellular differentiation and colorectal tumorigenesis.. Genes Dev.

[OCR_01157] Höhne M. W., Halatsch M. E., Kahl G. F., Weinel R. J. (1992). Frequent loss of expression of the potential tumor suppressor gene DCC in ductal pancreatic adenocarcinoma.. Cancer Res.

[OCR_01162] Kikuchi-Yanoshita R., Konishi M., Fukunari H., Tanaka K., Miyaki M. (1992). Loss of expression of the DCC gene during progression of colorectal carcinomas in familial adenomatous polyposis and non-familial adenomatous polyposis patients.. Cancer Res.

[OCR_01168] Klingelhutz A. J., Hedrick L., Cho K. R., McDougall J. K. (1995). The DCC gene suppresses the malignant phenotype of transformed human epithelial cells.. Oncogene.

[OCR_01180] Miller C. W., Aslo A., Tsay C., Slamon D., Ishizaki K., Toguchida J., Yamamuro T., Lampkin B., Koeffler H. P. (1990). Frequency and structure of p53 rearrangements in human osteosarcoma.. Cancer Res.

[OCR_01185] Miyake K., Inokuchi K., Dan K., Nomura T. (1993). Expression of the DCC gene in myelodysplastic syndromes and overt leukemia.. Leuk Res.

[OCR_01189] Mulligan L. M., Matlashewski G. J., Scrable H. J., Cavenee W. K. (1990). Mechanisms of p53 loss in human sarcomas.. Proc Natl Acad Sci U S A.

[OCR_01193] Nakajima-Iijima S., Hamada H., Reddy P., Kakunaga T. (1985). Molecular structure of the human cytoplasmic beta-actin gene: interspecies homology of sequences in the introns.. Proc Natl Acad Sci U S A.

[OCR_01198] Narayanan R., Lawlor K. G., Schaapveld R. Q., Cho K. R., Vogelstein B., Bui-Vinh Tran P., Osborne M. P., Telang N. T. (1992). Antisense RNA to the putative tumor-suppressor gene DCC transforms Rat-1 fibroblasts.. Oncogene.

[OCR_01203] Ookawa K., Sakamoto M., Hirohashi S., Yoshida Y., Sugimura T., Terada M., Yokota J. (1993). Concordant p53 and DCC alterations and allelic losses on chromosomes 13q and 14q associated with liver metastases of colorectal carcinoma.. Int J Cancer.

[OCR_01210] Persico M. G., Toniolo D., Nobile C., D'Urso M., Luzzatto L. (1981). cDNA sequences of human glucose 6-phosphate dehydrogenase cloned in pBR322.. Nature.

[OCR_01217] Pollice A. A., McCoy J. P., Shackney S. E., Smith C. A., Agarwal J., Burholt D. R., Janocko L. E., Hornicek F. J., Singh S. G., Hartsock R. J. (1992). Sequential paraformaldehyde and methanol fixation for simultaneous flow cytometric analysis of DNA, cell surface proteins, and intracellular proteins.. Cytometry.

[OCR_01224] Porfiri E., Secker-Walker L. M., Hoffbrand A. V., Hancock J. F. (1993). DCC tumor suppressor gene is inactivated in hematologic malignancies showing monosomy 18.. Blood.

[OCR_01229] Reale M. A., Hu G., Zafar A. I., Getzenberg R. H., Levine S. M., Fearon E. R. (1994). Expression and alternative splicing of the deleted in colorectal cancer (DCC) gene in normal and malignant tissues.. Cancer Res.

[OCR_01238] Thompson A. M., Morris R. G., Wallace M., Wyllie A. H., Steel C. M., Carter D. C. (1993). Allele loss from 5q21 (APC/MCC) and 18q21 (DCC) and DCC mRNA expression in breast cancer.. Br J Cancer.

[OCR_01247] Uchino S., Tsuda H., Noguchi M., Yokota J., Terada M., Saito T., Kobayashi M., Sugimura T., Hirohashi S. (1992). Frequent loss of heterozygosity at the DCC locus in gastric cancer.. Cancer Res.

[OCR_01252] Winkler K., Beron G., Delling G., Heise U., Kabisch H., Purfürst C., Berger J., Ritter J., Jürgens H., Gerein V. (1988). Neoadjuvant chemotherapy of osteosarcoma: results of a randomized cooperative trial (COSS-82) with salvage chemotherapy based on histological tumor response.. J Clin Oncol.

[OCR_01261] Yamaguchi T., Toguchida J., Yamamuro T., Kotoura Y., Takada N., Kawaguchi N., Kaneko Y., Nakamura Y., Sasaki M. S., Ishizaki K. (1992). Allelotype analysis in osteosarcomas: frequent allele loss on 3q, 13q, 17p, and 18q.. Cancer Res.

